# Partial AUCs in Long-Acting Injectables: Rationale, Challenges, Variability, Usefulness, and Clinical Relevance

**DOI:** 10.3390/pharmaceutics17010021

**Published:** 2024-12-26

**Authors:** Georgia Tsakiridou, Maria-Faidra-Galini Angelerou, Panagiotis Efentakis, Antonios Margaritis, Antigoni-Maria Papanastasiou, Lida Kalantzi

**Affiliations:** Pharmathen SA, 31 Spartis Str., 14452 Metamorfosi Attica, Greece; gtsakiridou@pharmathen.com (G.T.); maggelerou@pharmathen.com (M.-F.-G.A.); pefentakis@pharmathen.com (P.E.); amargaritis@pharmathen.com (A.M.); apapanastasiou@pharmathen.com (A.-M.P.)

**Keywords:** partial AUCs, bioequivalence, pharmacokinetics, variability, long-acting injectables, buprenorphine HCL, naltrexone, octreotide, lanreotide, exenatide, leuprolide

## Abstract

Regulatory authorities typically require bioequivalence to be demonstrated by comparing pharmacokinetic parameters like area under the plasma concentration-time curve (AUC) and maximum plasma concentration (C_max_). Because in certain cases, AUC and C_max_ alone may not be adequate to identify formulation differences in early and/or late segments of the dosing interval, partial AUCs (pAUCs) have been proposed as additional metrics to evaluate bioequivalence. Even though cut-off points for pAUCs are usually decided based on clinical relevance, the identification of the correct cut-off range remains elusive in many other cases and tends to contribute to increased pAUC estimate variabilities. The choice of meaningful cut-off points in pAUC estimates can be especially difficult in the case of long-acting injectable (LAI) products, where long dosing intervals and complex pharmacokinetic (PK) and pharmacodynamic (PD) profiles apply, but most importantly, because there is not always a clear PK/PD relationship established. In this communication, authors discuss the usefulness and challenges associated with the estimation of pAUCs in the development of generic LAI products through the review of six case studies under the lens of regulatory requirements from the two major authorities, namely the FDA and EMA.

## 1. Introduction

Bioavailability (BA) and bioequivalence (BE) assessments are routinely conducted during the development of new or generic drugs as part of the regulatory requirements for comparison of the rate and extent of drug absorption between products [1]. For drugs with systemic circulation, this is generally achieved by measuring drug concentrations in biological fluids over time in order to calculate pharmacokinetic parameters, such as maximum concentration (C_max_), area under the curve from zero to the last sampling point (AUC_0-t_), and area under the curve from zero to infinity (AUC_0-inf_). Two products are, generally, considered bioequivalent if the 90% confidence intervals (CIs) of the geometric mean ratios (test/reference) of relevant PK metrics (e.g., C_max_, AUC_0-t_, AUC_0-inf_) fall within the predefined bioequivalence limits (e.g., 80–125%).

Even though C_max_ and AUC are generally adequate to estimate the extent of absorption and overall exposure, they may not be sensitive enough to detect differences between two products that present major differences in the early [2,3] or the terminal [3] segment of the dosing interval. This is especially relevant for products where the shape of the PK profile influences clinical performance due to well-characterized pharmacokinetic/pharmacodynamic (PK/PD) relationships. In the context of this discussion, the major regulatory authorities worldwide (European Medicines Agency (EMA), U.S. Food and Drug Administration (FDA)) introduced the concept of partial AUC (pAUC), as a more sensitive approach for the investigation of BE. PAUCs are a measure of drug exposure during a specific time interval of interest, focusing only on a specific sub-area of the curve and providing insight into the drug behavior during critical phases of absorption, distribution, or elimination [4].

The first official mention of the need for pAUCs in regulatory guidelines for generic product development was in 1992, when Health Canada introduced the pAUC zero to reference product T_max_ (pAUC_Reftmax_) as an additional early exposure metric in cases where early exposure was important for the product’s efficacy [5]. Subsequently, in 2003, the FDA, in its general BA/BE guidance, recommended an early pAUC (defined as AUC truncated at the population median T_max_ for the reference product) for similar cases [6]. In 2011, the application of pAUCs was furthered to extended-release products with the issuance of FDA Product-Specific Guidelines (PSG) for methylphenidate and zolpidem [7,8]. The need for pAUCs in these cases was based on the products’ unique PK/PD characteristics that allowed for the identification of clinically relevant regions of the PK profile. Finally, in 2013, the FDA issued a draft guidance for industry, recommending the use of pAUCs for certain products as described in their PSGs and for “*modified-release products in which the different phases of release correspond to a clinical effect*” [9]. The FDA noted that the investigated partial area should be related to clinically relevant PD measures and that sufficient quantifiable samples should be collected to enable the adequate estimation of the metric.

In Europe, pAUCs were first introduced in 2013 in the context of the updated guideline for modified-release products [10]. In this guideline, for modified-release products where accumulation is likely (the AUC at the dosing interval covers less than 90% of the extrapolated AUC to infinity), the EMA requires a single and multiple-dose study for the demonstration of BE between test and reference products. In cases where low accumulation is expected, the requirement for the multiple-dose study can be waived, and BE can be investigated with the single-dose study alone. However, BE needs to be demonstrated not only for the traditional PK metrics (i.e., C_max_ and AUC_0-τ_) but also for additional pAUCs that will characterize the shape of the concentration versus time curve. EMA as a general rule recommends the use of half of the dosing interval (τ/2) as the cut-off point for pAUC calculation, while the suggestion for multiphasic modified-release products is that the cut-offs should follow the release phases. A summary of the requirements of pAUCs according to the FDA and EMA is presented in Table 1.

One of the first cases where pAUCs were proposed for the investigation of BE is the case of methylphenidate HCl products intended for the treatment of ADHD in children 6 years of age and older, adolescents, and adults up to 65 years. Even though methylphenidate HCl tablets (CONCERTA^®^, Janssen Pharmaceuticals, Beerse, Belgium) and capsules (METADATE^®^, UCB Inc., Brussels, Belgium) show comparable overall exposure and C_max_, the shape of their plasma profiles is substantially different, which seems to have an impact on clinical response [11,12]. More specifically, it was observed that depending on the PK profile, efficacy windows were different. Concerta^®^ provides greater symptom control in the early evening, while Metadate^®^ provides greater control in the morning. To ensure an equivalent onset response, both EMA and FDA propose the assessment of pAUC_0–3h_ (for fasting conditions) and pAUC_0–4h_ (for fed conditions) as primary PK metrics in the BE study, with 3 and 4 h representing T_max_ plus 2 standard deviations (SD). Accordingly, it is ensured that 95% of the immediate release component of the product has been eliminated [8,13,14]. Another example showing clinical relevance associated with a given pAUC, is Zolpidem tartrate extended-release tablets (Ambien CR^®^, Sanofi-Aventis, Paris, France), approved for the treatment of insomnia characterized by difficulties with sleep initiation. Zolpidem is formulated to achieve a rapid response followed by a prolonged effect by incorporating an immediate- and a controlled-release formulation element. Based on the originator’s clinical studies, most subjects (>90%) fell asleep 1.5 h after dosing, while fewer subjects were asleep at 1 h post-dose, and all subjects were asleep by hour 2. In addition, PK modeling showed that pAUC_0–1.5h_ is able to ensure an adequate early exposure and efficacy effect, along with C_max_ and total AUC [15]. In this context, pAUC_0–1.5h_ has been recommended by FDA guidance to be included as an additional PK metric for bioequivalence testing in studies using Ambien CR^®^ as the reference product [7].

However, the above conclusions related to pAUC clinical relevance have been made based on PK simulations and in vivo data of, mainly, orally administered products. The application of pAUCs in other formulation types and administration routes, showcasing increased PK and PK/PD complexity, is not yet fully realized. This is especially true for long-acting injectable products (LAIs) that are administered over long time intervals (1, 3, 6 months, etc.), have complex multiphasic PK profiles, and not always clear PK/PD relationships. Hence, the purpose of this communication is to discuss the usefulness and challenges associated with estimating pAUCs in the development of generic LAI products through the review of six case studies under the lens of regulatory requirements from the two major authorities: FDA and EMA. These products were selected due to the regulatory requirements around the application of pAUCs during their development. They also represent cases with varying rationales behind pAUC application (i.e., analytical, PK/PD, or other considerations).

## 2. Methodology

Data presented in this manuscript originated from published literature data, using WebPlotDigitizer 4.8 software for data extraction. Data were visualized and analyzed using the GraphPad Prism 9.0 software, and pAUCs were calculated for the determination of intersubject variability. PAUCs were calculated without the smoothing or curve fitting of the data, using the trapezoidal rule. SDs for AUC were calculated using the method described by [16]. Intersubject variability was calculated as CV% = SD/Mean × 100%.

## 3. Case Studies

### 3.1. Case Study: Buprenorphine

Buprenorphine HCl is a partial opioid agonist used in the treatment of moderate-to-severe opioid addiction. It is formulated as an extended-release injectable suspension, intended for monthly subcutaneous (SC) administration. Two products exist on the market: SUBLOCADE^®^ (Indvior Inc., Chesterfield, VA, USA) in the US and Buvidal^®^ (Camurus AB, Lund, Sweden) in the EU (as Brixadi^®^, Braeburn Inc., Plymouth Metting, PA, USA). Even though they are different in terms of formulation and available doses, they exhibit similar PK behavior and efficacy, allowing patients to switch from one product to the other in territories where both products exist, i.e., Australia [17].

**Figure 1:**
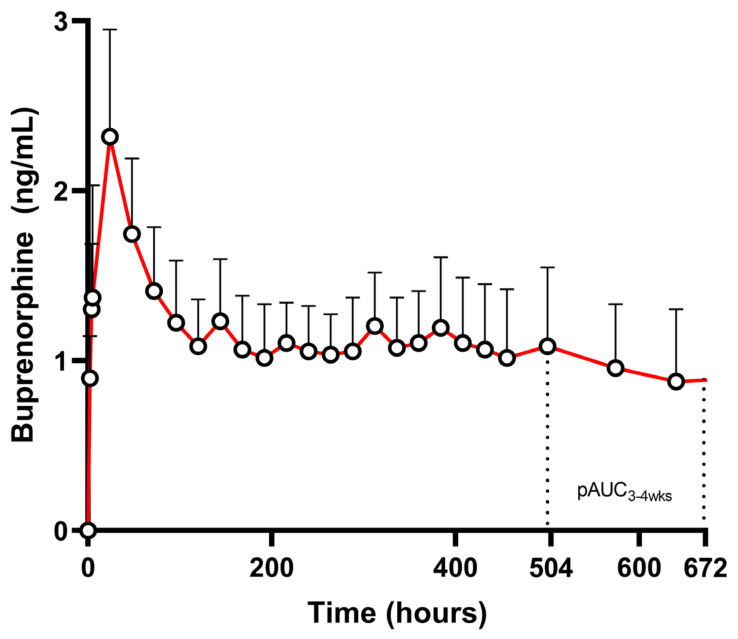
Pharmacokinetic profile (mean drug concentrations + SD) after single SC application of long-acting buprenorphine 200 mg in opioid-dependent treatment-seeking subjects (SUBLOCADE **^®^**) (n = 12). PAUC_3–4wks_ is highlighted (adapted from [20]).

The PK profile of these drug products consists of two main phases. First, there is an initial release phase marked by a single peak occurring between days 0 and 3 (the immediate-release phase). This is followed by a sustained-release plateau phase that lasts from days 3 to 28 (Figure 1) [18,19,20]. Buprenorphine is a partial μ-opioid receptor agonist with high-affinity and slow dissociation binding to μ-opioid receptors, thus suppressing opioid withdrawal, cravings, opioid use, and the effects of exogenous opioids [21,22]. Generally, several PK/PD models have been established in the literature for different buprenorphine products indicating correlations between plasma concentrations and clinical response, such as blocking of drug liking. More specifically, some studies have suggested that at least 50–60% μ-opioid receptor occupancy is required for suppression of opioid withdrawal and agonist symptoms. This has been correlated to varying plasma concentrations ranging from 0.7 to 3 ng/mL [22,23,24,25,26,27].

For the investigation of BE for these products, the FDA recommends C_max_, AUC_0-t_, and pAUC_3–4 weeks_. According to the FDA, for buprenorphine LAI products, C_max_ is deemed sufficient for confirming a comparable PK profile during the initial absorption phase [28]. Additionally, maintaining the average plasma minimum concentration (C_min_) during the plateau phase has been shown to be crucial for ensuring comparable clinical efficacy for a potential generic formulation, especially considering the PK/PD indications for a minimum plasma concentration to ensure efficacy. However, as C_min_ is a single-point metric typically occurring during the rapidly declining part of the concentration-time curve, its BE assessment becomes complicated due to increased variability. Hence, in order to compare the performance of innovator and generic buprenorphine LAI products, the FDA recommends the use of pAUC from 3 to 4 weeks (pAUC_3–4 weeks_) as a surrogate measure of the average plasma concentration over the final week following dosing along with the traditional PK metrics for the evaluation of BE [29].

The EMA, on the other hand, has not issued any relevant recommendation, letting buprenorphine HCl extended-release injectable products fall in the general guideline for modified-release products. In that regard, given that accumulation is expected [30], a multiple-dose study cannot be waived, and thus pAUCs are not required [10]. However, in case that accumulation would be negligible, the pAUC_3–4 weeks_ could not be supported. Conversely, pAUCs assessment could be proposed based on the EMA recommendation for cut-off points at the half of the dosing interval (pAUC_0–14d_ and pAUC_14–28d_) or based on the PK profile of the product, which shows an almost completed initial burst after day 3 (pAUC_0–3d_ and pAUC_3–28d_). However, based on population PK analysis, there seems to be high interindividual variability regarding the absorption phase kinetics, owing to formulation and/or physiological characteristics [18,19]. More specifically, after a single-dose administration of buprenorphine 192 mg in healthy volunteers, pAUC_0–3d_ shows an intersubject variability of 59.5%, while pAUC_0–14d_ shows an intersubject variability of 37.14% (Table 2). This highlights that earlier cut-off points are indeed related to increased intersubject variability, owing to either physiological and/or formulation-specific absorption-related processes. Even though direct extrapolation for intrasubject variability is not possible solely based on these data, high intersubject variability could raise concerns regarding the investigation of BE. This could be especially relevant if the high intersubject variability is due to formulation-related implications, thus necessitating a different study design and/or a possible increase in sample size. For example, an intersubject variability of ~60% implies an intrasubject variability equivalent of >30%, which then points towards a replicate BE study design instead of a parallel or a crossover.

### 3.2. Case Study: Naltrexone

Naltrexone is an opioid antagonist formulated as an LAI product intended for intramuscular (IM) monthly injection (Vivitrol^®^, Alkermes, Inc., Waltham, MA, USA). It is indicated for the treatment of alcohol dependence and for the prevention of relapse to opioid dependence following opioid detoxification [31]. This extended-release form is designed to improve treatment adherence, addressing the issue of patient noncompliance that can reduce the effectiveness of oral naltrexone [32].

**Figure 2:**
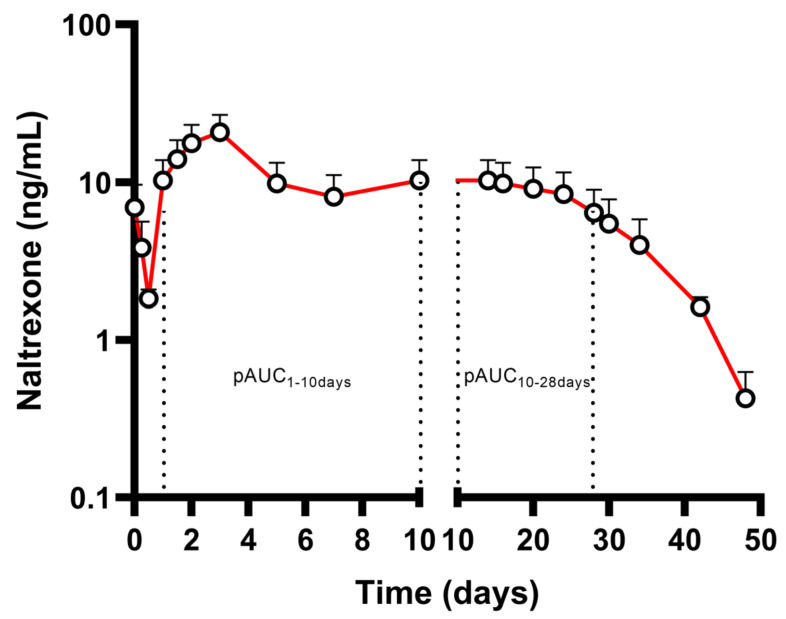
Pharmacokinetic profile (mean drug concentrations in the logarithmic scale + SD) after a single application of long-acting naltrexone 380 mg (Vivitrol^®^) in healthy volunteers (n = 10). The X-axis is segmented to highlight the behavior of the formulation between Days 1–10. PAUC_1–10days_ and pAUC_10–28days_ are highlighted (adapted from [34]).

After IM injection, naltrexone plasma concentrations exhibit a transient initial peak, which occurs approximately 2 h after injection, followed by a second, larger peak observed approximately 2–3 days later. Beginning approximately 14 days after dosing, concentration slowly declines, retaining measurable levels for greater than 28 days. The PK profile after a single-dose IM administration of naltrexone 380 mg (Vivitrol^®^) is depicted in Figure 2 [33,34]. Naltrexone exerts its effect by blocking the μ-opioid receptor and by being a weaker antagonist of the kappa and delta-opioid receptors, suppressing the effect of opioids and preventing opioid intoxication and physiological dependence on opioid users. Naltrexone also modifies the hypothalamic–pituitary–adrenal axis to suppress ethanol consumption [35]. The minimum effective therapeutic plasma concentration for naltrexone is not well understood. Some studies have shown that plasma concentrations of approximately 1–2 ng/mL are sufficient to antagonize heroin-induced effects [36,37,38,39], while the relevant concentration range for the effective treatment of alcohol dependence has not been definitively established. Interestingly, it has been suggested that naltrexone may continue to block brain opioid receptors even when measurable plasma concentrations of the drug are no longer detectable [40].

Based on the above, the FDA recommends two pAUCs as primary PK parameters in the investigation of BE of generic naltrexone LAI products, along with the traditional PK metrics to ensure similar rate and extent of release [41]. These pAUCs include an early (pAUC_1–10d_) and a late exposure (pAUC_10–28d_) metric, with 10 days post injection being the cut-off point (Figure 2). The two pAUCs after the administration of a single dose of naltrexone 380 mg (Vivitrol^®^) show intersubject variabilities of 39.64% and 45.22%, respectively (Table 3). pAUC_0–1d_ has not been included in this recommendation, potentially due to the miniscule exposure, along with the increased intersubject variability of the metric (~50%) and the complications of limited sampling in the first day after dosing [34]. Nevertheless, it can be estimated that most of the effect of the initial two peaks is eliminated by day 10; hence, it is an appropriate cut-off for the estimation of early exposure. However, since naltrexone’s therapeutic effect is reliant on a minimum blood concentration, it could be argued that this metric is a measure of safety rather than efficacy. It has been reported in the literature that high naltrexone blood levels could be correlated with severe adverse reactions and hepatic toxicity [42,43,44,45]. It is worth mentioning that based on the originator’s data, pAUC_0–7d_ was also investigated as a sensitive metric but was not selected by the FDA to be incorporated in the product-specific guideline. Finally, the late exposure pAUC can be seen as a safeguard for the average plasma concentration over the later part of the dosing interval, which is extremely important for the efficacy of these products.

The EMA has not released any relevant guidelines as no naltrexone LAI products are marketed in Europe. However, since only minimal accumulation is expected for this product [19], the EMA would require at least two pAUCs in order to characterize the shape of the blood concentration versus time curve. In that respect, we would be of the opinion that the pAUCs as proposed by the FDA would also be acceptable by the EMA as they seem to take into account the two phases of release of the product, corresponding to the initial absorption phase and the late partial area related to the clinically meaningful C_min_.

### 3.3. Case Study: Octreotide

**Figure 3:**
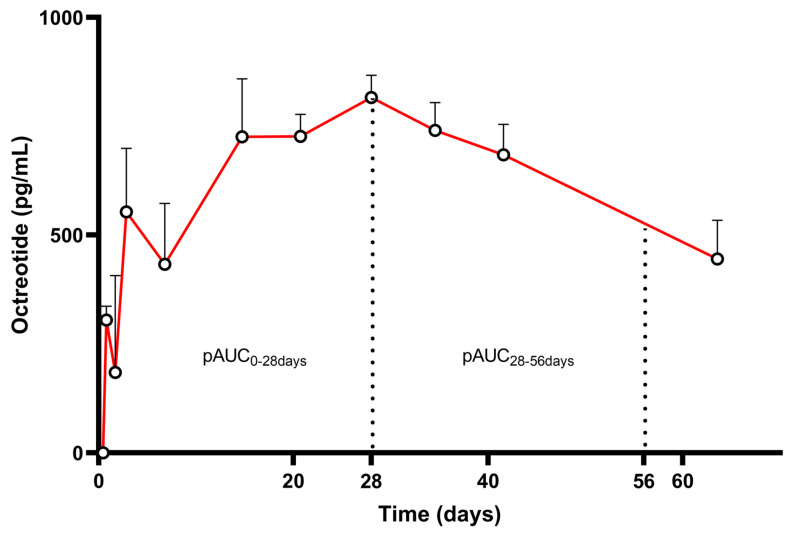
Pharmacokinetic profile (mean drug concentrations + SD) after single application of long-acting octreotide 20 mg (Sandostatin LAR^®^) in patients with acromegaly (n = 33) (Adapted from [50]). pAUC_0–28d_ and pAUC_28–56d_ are highlighted.

Octreotide is a somatostatin analog formulated as an LAI product, administered via deep IM injection every 28 days (Sandostatin LAR^®^, Novartis Pharmaceuticals Corporation, East Hanover, NJ, USA), used for the management and treatment of acromegaly and thyrotrophinomas [46]. The single-dose PK profile for this product is characterized by three main phases: (i) a transient initial peak within 1 h after a single IM administration, followed by a progressive decrease to a low undetectable level within 24 h; (ii) a progressive decrease to a low undetectable level within 24 h; and (iii) the main release phase in which octreotide concentrations increase again to reach a plateau around day 14 (about 2–3 weeks post-injection), maintained during the following 3 to 4 weeks. Subsequently, a decline period of 6 weeks follows, leading to measurable octreotide concentrations up to 9 weeks [47]. Two PK profiles after a single application of long-acting octreotide 20 mg (Sandostatin LAR^®^) in patients with acromegaly are shown in Figure 3. The insert represents the mean PK profile of the two single-dose studies.

Octreotide exerts its effect by inhibiting the release of pituitary and gastroenteropancreatic hormones, i.e., serum growth hormone (GH) and insulin-like growth factor 1/somatomedin C (IGF-1), which are hyperexcreted in GH-releasing hormone-secreting adenoma promoting acromegaly. Unlike somatostatin, octreotide inhibits GH preferentially over insulin, and its administration is not followed by rebound hypersecretion of hormones [48]. Following administration of the octreotide LAI, the pattern of growth hormone (GH) secretion, regardless of dose, shows an initial suppression lasting 8 to 12 h. GH levels return to nearly pre-injection levels on days 2, 3, and 7, and from days 14 to 42, GH secretion is completely suppressed [49]. This behavior follows the PK pattern of octreotide release during the three phases of in vivo performance.

In the case of octreotide, the FDA and EMA have similar approaches, with both recommending that only a single-dose study in healthy volunteers should be sufficient to demonstrate BE (PSGs). Given that octreotide LAI products show accumulation in vivo, the EMA justified the waiver of the multiple-dose study based on the feasibility issues along with the predictability of overlapping profiles [51]. Both agencies require the study to last 56 days (twice the dosing interval of 28 days) to account for the continued release and to include two pAUCs: one up to day 28 and one from day 28 to 56 (pAUC_0–28d_ and pAUC_28–56d_). Concentration at the end of the dosing interval (C_τ_) is also proposed by EMA as an alternative PK metric in order to ensure that comparable and therapeutically relevant concentrations are observed until that point. C_τ_ is also an excellent parameter with strong discriminative power to assess steady-state behavior, and since it is not in the curve region of rapidly declining concentrations, it is not expected to pose severe challenges for BE investigation, variability-wise. Additionally, pAUC_0–24h_ and C_max_ within each pAUC region are proposed by EMA as secondary parameters to elucidate burst release absorption and the extent of release within each pAUC.

By segmenting the total AUC into 0–28 and 28–56 day periods, the majority of the release profile is captured. Since the majority of exposure is usually observed from day 7 onwards, with only minimal exposure seen in the first peak (1/100), pAUC_0–28d_ can be reasonably considered a sensitive enough metric to ensure equivalent therapeutic exposure throughout the whole interval. This is particularly emphasized by the strong PK/PD relationship observed for this product, with GH secretion directly correlating with octreotide concentrations. Interestingly, based on PK modeling [52], it has been described that the release from octreotide LAI is not uniform, with potential sub-populations present in the population PK curve. Consequently, the mean PK profile may not fully represent individual characteristics. This is further elucidated by the fact that the same product, when given to patient populations, can give rise to slightly different PK profiles (Figure 3, blue and red profiles). In this case, defining more segmented pAUCs to better capture each individual release phase of the product would result in metrics with unmanageable variability and limited clinical relevance. Finally, the late exposure pAUC_28–56d_ can be seen as a metric to ensure similarity in steady-state profiles, quantifying the remaining exposure after the dosing interval.

### 3.4. Case Study: Lanreotide

**Figure 4:**
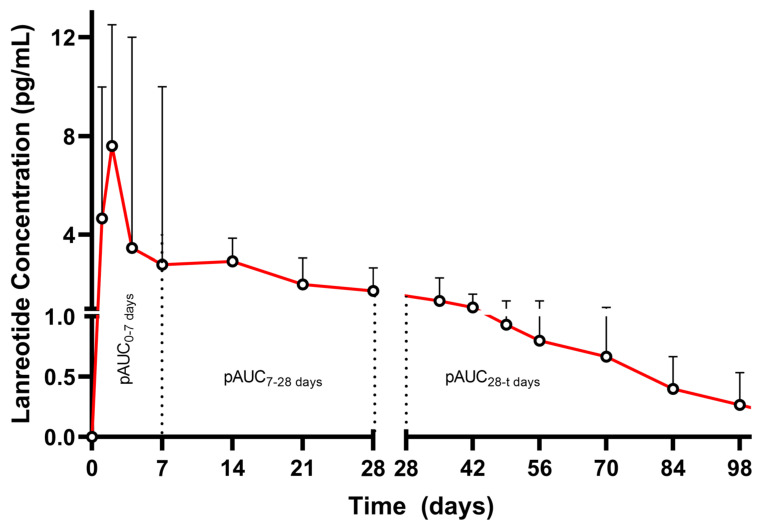
Pharmacokinetic profile (mean drug concentrations + SD) after a single application of prolonged-release lanreotide 90 mg (Somatuline Autogel^®^) in healthy volunteers (n = 13). The X-axis and Y-axis are segmented to show the profile of the product in the initial phase (Days 0–28). pAUC_0–7days_, pAUC_7–28days_, and pAUC_28-t_ are highlighted. Insert represents the full profile without axis scaling (adapted from [60]).

Lanreotide is a somatostatin analog formulated as an LAI product, administered via deep SC injection every 28 days (Somatuline^®^ depot, Ipsen Pharma SAS, and Somatuline^®^ Autogel, Ipsen Ltd., London, UK), used for the treatment of acromegaly and gastroenteropancreatic neuroendocrine tumors [53]. The release profile of lanreotide is characterized by an initial burst release on the first day of administration that determines the C_max_, followed by a sustained release (Figure 4). This behavior is conditional to the peptide self-assembling into a gel under specific conditions. Due to dense packing, peptide filaments form a semi-solid gel via noncovalent bonding. The assembly is reversible, allowing controlled drug release [54,55,56].

Lanreotide is a peptide inhibitor of multiple endocrine, neuroendocrine, and exocrine mechanisms, and it reduces GH secretion and Insulin Growth Factor-1 (IGF-1). It has been shown to achieve 90% inhibition of GH; an average drug level of 1000 ρg/mL would be needed in patients with acromegaly [47,57].

The case of lanreotide LAI is unique, as the release is dependent solely on the folding and unfolding of the API. As such, in order to support a generic application, the FDA suggests either a biowaiver approach based on the physicochemical sameness between the originator and the generic API or a conventional in vivo BE study [58]. No pAUCs are recommended since clinically relevant individual release phases are difficult to distinguish within the dosing interval. In addition, the PK/PD relationship of the product stipulates the need to retain concentration levels above a threshold for efficacy rather than correlate to specific curve shape characteristics [47,57].

On the other hand, the EMA suggests either a biowaiver approach or only a single-dose in vivo BE study, acknowledging the feasibility issues for a multiple-dose study in the intended population (despite accumulation being expected for this product) [59]. Multiple pAUCs are included as primary metrics in the study requirements for BE assessment (pAUC_0–7d_, pAUC_7–28d_, and pAUC_28d-t_). In principle, these pAUCs are meant to characterize the different phases of the PK curve of the product.

Still, as evident also in Table 4, it is possible that individual concentrations/profiles are quite variable. More specifically, after a single dose of lanreotide 90 mg LAI (Somatuline Autogel^®^) (n = 10), intersubject variability values for pAUC_0–7d_, pAUC_7–28d_ and pAUC_28d-t_ are 52.71%, 64.11%, and 108.90%, respectively. Intersubject variabilities above ~45% indicate an intrasubject equivalent of >30%, and then the appropriate study design to demonstrate BE is the replicate design. Still, the replicate design requires multiple administrations, an approach that was characterized by EMA as non-feasible.

### 3.5. Case Study: Exenatide

**Figure 5:**
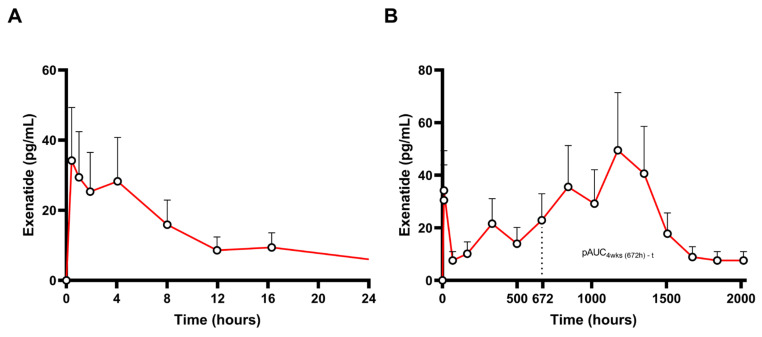
Pharmacokinetic profile (mean drug concentrations + SD) after a single application of a 2.5 mg subcutaneous dose of long-acting exenatide injectable (Bydureon^®^) during the first 24 h post dose (**A**) and 12 weeks (2016 h) post dose (**B**). pAUC_4 wks-t_ is highlighted (adapted from [63]).

Exenatide is a synthetic form of a naturally occurring GLP-1 receptor agonist, administered once-weekly SC as a long-acting injectable (Bydureon^®^, AstraZeneca Pharmaceuticals LP/AstraZeneca AB) for the treatment of type 2 diabetes mellitus [61]. Upon administration of a single dose of the exenatide LAI product, exenatide is gradually released from the microspheres over a period of approximately 10 weeks [62]. Initially, there is a release of surface-bound exenatide (burst effect of 1–2% of exposure), followed by a slow release from the microsphere formulation, resulting in two peaks in plasma exenatide levels: within week 2 and between weeks 6 and 7. The single-dose PK profile of a 2.5 mg subcutaneous dose of long-acting exenatide injectable (Bydureon^®^) up to 12 weeks can be seen in Figure 5 (adapted from [63]). Figure 5A depicts the PK profile of the first 24 h post dose, while the whole PK profile is shown in Figure 5B.

Exenatide binds to the GLP-1 receptor and lowers glucose levels through several mechanisms, such as delaying gastric emptying, reducing glucagon levels, enhancing feelings of satiety, and promoting glucose-dependent insulin [61,64]. A minimal effective concentration of 50 pg/mL has been described in the literature [62,65].

Even though the exenatide LAI product is administered weekly, concentrations are detectable for up to 10 weeks after a single administration. This release pattern allows gradual exposure escalation, leveraging the accumulation of the product and not requiring dose titration. More specifically, after 6 or 7 weeks, a mean plasma concentration of roughly 300 pg/mL is sustained with weekly doses, indicating that steady-state levels have been reached. This gradual progression to a steady state appears to enhance tolerability, as exenatide LAI is associated with less frequent nausea compared to oral exenatide products. The slow rise in plasma exenatide concentrations minimizes the need for gradual dose escalation, which is often necessary with shorter-acting GLP-1 receptor agonist formulations [66].

Based on this, the FDA recommends incorporating a late pAUC metric (i.e., the area under the curve from week 4 to the final sampling point, pAUC_4w-t_) as an additional pharmacokinetic metric in the assessment of BE in a single side [67]. It has been proposed that this metric aims to characterize and safeguard the sameness of accumulation between products, which will in turn control steady-state attainment during weekly administration [68]. If the investigation of BE in a single-dose study is not possible, a multiple-dose study in patients is alternatively proposed [67].

In contrast, as per EMA guidelines, given that clinical practice would rely on the accumulation of the product, a single- and a multiple-dose study would be needed. Interestingly, the EMA recommends the inclusion of the maximum plasma concentrations during the initial and extended-release phases (referred to as C_max,1_ and C_max,2_, respectively), alongside AUC_0-t_ and AUC_0–∞_ for the assessment of BE in the single-dose study [69].

### 3.6. Case Study: Leuprolide

Leuprolide is a gonadotropin-releasing hormone (GnRH) agonist used for the management of endometriosis and uterine leiomyomata (also known as uterine fibroids) as well as the treatment of central precocious puberty in children and advanced prostate cancer [70,71,72]. Leuprolide is available as varying LAI formulations for IM and/or SC administration, such as ready-for-use implants, ampoules, or vials with powder and solvent for prolonged-release suspension (or solution) for injection and powder and solvent for prolonged-release suspension for injection in pre-filled syringes.

**Figure 6:**
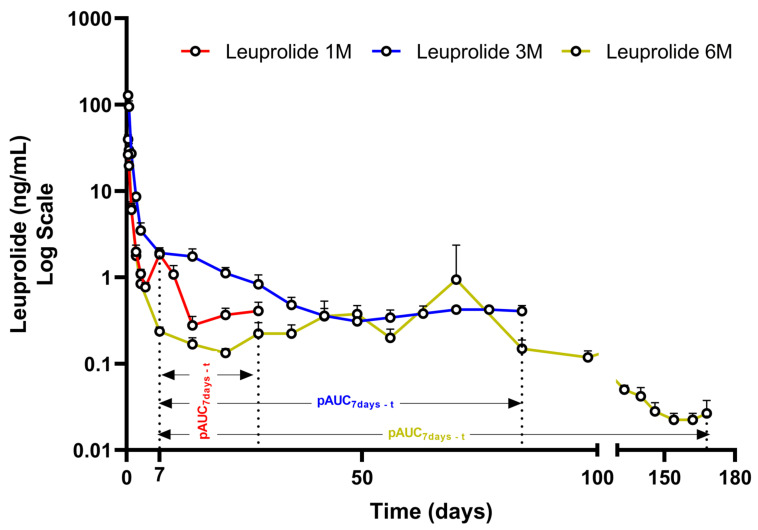
Release pattern of leuprorelin as median serum concentrations (in the logarithmic scale) for three depot formulations (Eligard^®^) injected subcutaneously monthly (7.5 mg, red, n = 20), at 3-month intervals (22.5 mg, blue, n = 22) or at 6-month intervals (45 mg, yellow, n = 27) (Adapted from [76]). pAUC7_days-t_ is highlighted for both formulations.

**Figure 7:**
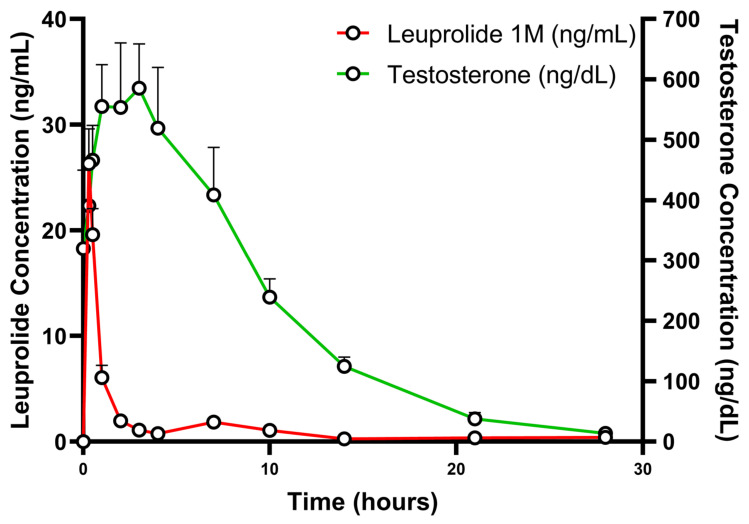
Typical shape of mean drug concentration-time curve of leuprolide (mean concentration + SD, red) and corresponding testosterone concentration (mean concentration + SD, green) after the administration of the 1-month formulation, 3.75 mg (n = 24) (adapted from [76]).

Leuprolide formulations are designed for sustained release over extended periods (typically 1, 3/4, or 6 months) using biodegradable delivery systems. The release of Leuprolide is multiphasic with an initial burst, a lag phase when release from the formulation slows down, followed by a plateau phase of steady Leuprolide concentrations (Figure 6). This multiphasic PK profile of leuprolide is linked to its efficacy. The burst phase relates to the sufficient binding between leuprolide and GnRH receptors, which causes a “flare up” in the production of luteinizing hormone (LH) and follicle-stimulating hormone (FSH), leading to an increase in steroidogenesis in ovaries and testes, thus, resulting in increased estrogen in females and increased testosterone and dihydrotestosterone in males. Ultimately, during the sustained concentration phase, continuous exposure to leuprolide causes GnRH receptor desensitization, resulting in decreased sex hormone synthesis and secretion, with estradiol falling to postmenopausal concentrations in women and testosterone falling to “castrate levels” in men [73]. Testosterone response after the administration of the leuprolide 1-month formulation is depicted in Figure 7.

As a result of the complex PK profile of leuprolide products and the importance of the PK curve shape in determining efficacy, additional alternative metrics are required to ensure bioequivalence between products. The FDA recommends evaluating the pAUC_7d-τ_ (the exposure from day 7 to the end of the dosing interval) along with the traditional metrics [74], based on clinical significance. More specifically, the FDA proposes that the most clinically significant phase of leuprolide release is the plateau phase, which typically begins on day 7 post-administration, as continuous leuprolide concentration is associated with receptor desensitization and gonadal suppression [75]. In contrast, leuprolide long-acting formulations fall under the EMA’s modified-release guideline, which specifies that when a low accumulation is anticipated, the shape of the single-dose PK curve must be characterized using scientifically justified pAUC metrics (early and terminal pAUC) [10].

However, late pAUCs in the case of leuprolide could be challenging. It is important to note that in prostate cancer patients, leuprolide concentrations are quite low, with the majority of them being in the vicinity of the lower limit of quantification (LLOQ). This analytical region is usually difficult with a lot of inherent variability [77]. This is normally not an issue, as in most cases blood concentrations close to LLOQ are limited during the end of the dosing interval. In the case of leuprolide, however, this region can last from 20 days to almost 6 months, depending on the dosing strength. When considering that the mean leuprolide plateau concentrations are almost parallel to the time axis (Figure 7) and that high analytical variability is expected for individual samples, it is evident that small changes in individual concentrations could cause inflated pAUC variability. This is especially true for cases where the majority of exposure comes from the erosion phase, which is an inherently variable process (i.e., pAUCs with cut-off points initiating once the burst and lag effect has concluded, i.e., past 72 h). Notably, intersubject variability for pAUC_24h-t_, pAUC_48h-t_, pAUC_72h-t_, pAUC_5d-t_, and pAUC_7d-t_ after the administration of the 1-month leuprolide 3.75 mg formulation is 19.59%, 19.07%, 18.34%, 60.87%, and 62%, respectively (Table 5). Given that this phenomenon relates to analytical procedures and not physiology or drug-related complications, it is expected to affect not only intersubject but also intrasubject variability. Hence, the inclusion of late pAUCs in the investigation of bioequivalence of leuprolide depot products can be challenging, with high intrasubject variabilities complicating sample size requirements and study design. An intersubject variability of 62% indicates a need for a replicate BE study design, which will allow widening of acceptance criteria for BE.

## 4. Discussion

pAUCs have been shown to be a powerful tool in revealing formulation differences at specific points of the dosing interval, especially for oral formulations with relatively simple PK profiles and clear PK/PD relationships. The main challenge behind the proper use of pAUCs is the selection of appropriate cut-off points for their calculation [13,27,79]. Usually, cut-off points are proposed based on either the PK characteristics of the product, i.e., fast- and slow-release phases that can be distinguished using the T_max_ of each phase as a cut-off point, or the product’s PK/PD relationship that would allow the identification of a point within a clinically relevant region [80]. The first approach, however, is not straightforward—not even in the case of simple oral products—with various approaches being proposed: (i) cut-off using the population median of T_max_ of the reference product [6], (ii) cut-off using the T_max_ of the reference formulation calculated for each subject [5,81] or (iii) the cutoff time point being the earlier of the two T_max_ values [82,83]. On the other hand, the second approach is strongly endorsed by all stakeholders, including industry, academia, and regulatory bodies, which underlines the need for determining cut-off points for pAUCs through a mechanistic and holistic understanding of the product requirements.

Still, it is evident that the selection of cut-off points can impact the test/reference mean ratio and influence the failure or success of a BE study. This notion has been investigated in two different publications for orally administered products: one from Brazil’s regulatory agency, ANVISA [84], and one from one of the biggest contract research organizations (CROs) of North America, AltaSciences [85].

More specifically, Soares et al., [84], collected 117 successful BE studies that were referenced in the registration of 59 different generic products approved by ANVISA since 2008 and calculated early and late pAUCs, pAUC_0-τ/2_ and pAUC_τ/2-t_, respectively. Additionally, the mean test/reference ratios of these metrics, along with their intrasubject variabilities, were calculated. For 41% of the studies, the 90% CIs for the early and late pAUCs mean test/reference ratios failed to meet the BE criteria and were outside the acceptance range of 80–125%. Similarly, Boily et al. [85] collected 53 pivotal two-treatment crossover single-dose studies in healthy volunteers, using orally administered modified-release products (mostly analgesics 33.96%, antidepressants 18.87%, and urologicals 9.43%). All these studies were considered successful for the traditional BE metrics, C_max_ and AUC_t_. Early (pAUC_Refmax_, pAUC_0-τ/2_) and late pAUCs (pAUC_τ/2-t,_ pAUC_τ/2-τ,_ pAUC_τ/2-inf_), and their mean test/reference ratios and intrasubject variabilities were then calculated post hoc to investigate the effect of the different cut-off points. Interestingly, almost half of the studies tested failed to meet the BE criteria for the early pAUC_Refmax_ (46.96%).

Collectively, these results show that the investigation of BE for the pAUC metrics can be challenging. Otherwise, passing studies can fail for these metrics either due to formulation differences between test and reference in the early phase or due to an insufficient sample size due to the increased variability [85]. Generally, it has been described that early pAUCs, for both immediate- and extended-release oral formulations, tend to have increased variabilities compared to the other conventional metrics of BE [86,87,88,89]. This can be attributed to the fact that pAUCs usually concern a limited region of exposure, even in the span of 30–90 min for some oral products. In this context, small variations in drug absorption/exposure can have a magnified effect on the calculation of the metric. More specifically, earlier cut-off points result in higher variability compared to truncation to later cut-off points, due to variations in the absorption process [85]. Additionally, it should be noted that even though the failed studies are not correlated to a specific drug class, it is possible to infer that early partial AUCs do not hold clinical significance for all tested drug classes. Consequently, misalignment of cut-off points with clinical relevance may introduce bias by potentially rejecting products that do not pass BE for clinically irrelevant pAUCs [84,85]. For example, the effect of traditional antidepressants, included in the study of Boily et al. [86], is famously not related to a rapid onset [82] as is the case of analgesics, but rather on a gradual attainment of steady state. Consequently, it should be considered whether early pAUCs, such as pAUC_Refmax_, are an appropriate metric for such an investigation and ponder on the possible implications this entails for generic product availability, given the identified variability implications.

Currently, the majority of what we know in terms of application and limitations of pAUCs is derived from in vivo and modeling data of oral products. Hence, the applicability of the metrics for the investigation of BE for other types of products and other routes of administration has not been solidified. Even though relevant EMA guidelines propose the use of pAUCs in complex products in a similar way to oral modified-release products [10], the actual applicability of the metrics remains a review issue and is on a case-by-case basis, without explicit guidance regarding their use in more complex products.

In this context, pAUCs are being increasingly proposed by regulatory authorities to support the generic development of LAIs. LAIs usually showcase months-long dosing profiles along with complex PK characteristics with evidence that the shape of the PK profile during certain time windows may affect clinical performance. In this context, pAUCs can be very powerful tools in characterizing complex PK behavior and investigating PK/PD similarity of clinically relevant regions of the dosing interval. However, it is vital that the correct windows are chosen to represent therapeutic effects while at the same time acknowledging considerations for potential inflated variability of the metrics.

In our perspective, the usage of pAUCs in the investigation of BE for LAI products has varying applications, ranging from a tool to mitigate the problems associated with C_min_ evaluation in BE, in the case of buprenorphine LAI products, to simplifying complex PK profiles with the inclusion of clinically relevant timepoints in the case of octreotide LAI. However, there are cases where appropriate cut-off points are not easily identifiable. For example, in the case of exenatide products, the proposed pAUC_4w-t_ was shown to be less sensitive than other metrics in identifying formulation differences between exenatide products in the context of a PK modeling approach [67]. More specifically, results from this analysis demonstrated that pAUC_4w-t_ exhibited similar sensitivity to C_max,1_ and C_max,2_, while later pAUCs, closer to T_max,2_, presented higher. Most notably, pAUC_7w-t_ showed the highest sensitivity to formulation differences. These results further highlight that the choice of cut-off points for pAUCs is of the utmost importance to achieve appropriate use.

Additionally, there are cases where the proposed pAUCs are of higher risk for increased variability compared to traditional PK metrics, which could become a major challenge during generic development. In general, intrasubject variability (or intersubject variability in parallel designs) is the driving force behind empirical and statistical sample size calculations for BE studies [89]. The effect of variability on the sample size required to demonstrate BE using the two most common study designs (parallel and crossover) is shown in Table 6. For instance, in a crossover study where the intrasubject variability for traditional BE metrics is 20%, while the intrasubject variability for pAUC is 30%, the sample size needed doubles from 37 to 79. It is evident that increased pAUC variability could result in a study with an insufficient sample size, if not considered a priori or could create feasibility issues if proven unmanageable, as in the case of recruitment challenges based on the intended population. In order to circumvent this, regulatory authorities also provide the possibility for BE limit widening in the case of high pAUC intrasubject variability, which can significantly decrease sample size requirements [9,90]. However, this is contingent on the appropriate characterization of the intrasubject variability for the reference formulation via two consecutive administrations of the product. This is usually facilitated by study design options, such as the partial or fully replicate crossover designs, where each subject is their own control by receiving the reference formulation twice. Even though EMA and FDA have slightly different statistical approaches for the calculation of widened BE limits, both agencies recognize products showing intrasubject variability of more than 30% as highly variable drugs, necessitating BE limit widening.

More specifically, as per EMA guidelines and best practices [90,91], the preferred method for statistical analysis and BE limit calculation is Method A, which is a fixed effect model (PROC GLM). EMA also describes Methods B and C, with Method B being a mixed effect model (PROC MIXED) that is a slight modification of Method A by specifying subject as a random effect and Method C being a mixed effect model (PROC MIXED) following FDA’s statistical analysis guidelines for the fully replicate study design [9,86]. This model allows a different subject effect for each formulation (i.e., a subject-by-formulation interaction) and therefore has five variance terms (within subject for reference, within subject for test, between subject for test, between subject for reference, and covariance for between subject test and reference—the last three are combined to give the subject×formulation interaction variance component.). This model will provide the same point estimate as methods A and B if all subjects provide data for all treatment periods. However, it will generally give wider confidence intervals than those produced by methods A and B. It is worth mentioning that the FDA proposes acceptance BE limits for a fully replicate design for highly variable drugs (i.e., S_WR_ ≥ 0.294) in a different way than the EMA. More specifically, the 95% upper confidence bound for ${\left({\overset{ˉ}{\Upsilon }}_{T}-{\overset{ˉ}{\Upsilon }}_{R}\right)}^{2}-\vartheta S_{WR}^{2}$ must be ≤0, and the point estimate of the test/reference geometric mean ratio must fall within [0.80, 1.25] [9,92]. Finally, the FDA also proposes the use of a fixed effect model (PROC GLM) for the statistical analysis of data from a partially replicated design. Based on the above, sample size calculation and BE limits for a fully replicate crossover study design are presented in Table 7, contingent on intrasubject variability and regulatory authority preferred practices.

One such case with high variability is the early exposure metrics of lanreotide LAI products due to the increased intersubject variability of the EMA proposed pAUCs [47,93,94]. As per EMA’s recommendation, the BE should be investigated in a parallel design setting in healthy volunteers due to the scarce availability of patients, long dosing interval, and safety precautions for the population [59]. However, it becomes evident that intersubject variability of pAUCs will eventually have to drive the study design and sample size calculation of the BE study. Variability of up to 100% (Section 2, Lanreotide Case Study) requires a sample size of 619 evaluable volunteers in a parallel design setting (assuming T/R ratio of 90–111%, 80% power). Such sample size requirements render a BE study unfeasible in the context of generic product development of an orphan drug, due to the long recruitment and clinical phases, which would not only compromise data integrity but also require extensive cost allocation.

Another such case is the late exposure metrics of the leuprolide LAI products, which show increased variability, potentially due to the analytical variability of the plateau phase concentrations (Section 2, Leuprolide Case Study). Investigation of BE for leuprolide products needs to be performed in patients for safety reasons due to the mechanism of action of the drug, which suppresses the secretion of gonadal hormones, promoting unacceptable adverse reactions for the healthy population. This complicates the situation as the intended patient population of advanced prostate cancer patients is considered high to very high risk and is a sensitive population with a number of comorbidities [95] and recruitment challenges [96,97]. Hence, a parallel study design that would require approximately 200 evaluable subjects, considering intersubject variability of ~50% (Table 6), would be exceptionally challenging. On the other hand, a fully replicated design that would allow the widening of BE limits would also be demanding, due to the fact that a washout period to account for a carryover between test and reference products is not an option for ethical reasons.

The above LAI products serve as an example of the potentially challenging nature of pAUC determination, factoring in the variability implications in a case-by-case scenario, according to specific drug needs. They are also prime examples of how pAUCs, if determined without any consideration for variability, could pose serious implications for the generic availability of such products by creating unfeasible study requirements. Of note, none of the GnRH agonist LAI products (the drug class of leuprolide) have available generics on the market, even though they were first marketed in the late 1980s [98]. In addition, the first octreotide generic product in the world was launched in October of 2024 in the US, more than 35 years after the introduction of the reference product Sandostatin^®^ LAR Depot in 1988 [99]. The importance of this is especially highlighted when considering the great diligence the FDA has put into highlighting and addressing the hurdles in complex generic product approval in recent years, from study design to dossier review and approval [100,101,102].

Finally, it is evident that there is a discrepancy in pAUC cut-off recommendations between the two major regulatory authorities, the FDA and EMA, that sometimes leads to proposing different pAUCs with different variabilities for the same product, depending on the territory. Interestingly, this different approach sometimes dictates a need for different clinical strategies for product approval, especially in cases where the different pAUCs are correlated with higher than 30% intrasubject variability in one territory but not the other. However, intrasubject variability should have been an inherent product characteristic, irrespective of pAUC cut-off point determination. This notion leads to implications for product approval across different regions based on regulatory requirements rather than actual product characteristics.

Even though this manuscript is aimed as a literature review and critical discussion of the applicability and challenges of pAUCs in the development of generic LAI products, it is also bound by data availability in the public domain. One of the main limitations of this publication is the absence of statistical investigation of intrasubject variability of the additional pAUCs. However, for such an analysis, individual concentration data would be needed rather than mean PK profiles, which are exceptionally rare in the literature.

Overall, this communication can serve as a discussion stimulus regarding the use of proper pAUCs to support generic product development for LAIs. Generally, it seems that the problem of identifying appropriate pAUCs is slowly maturing within industry and academia, with PK and PK/PD modeling being, recently, used in order to propose sensitive pAUCs for the development of Paliperidone and exenatide LAI products [68,103]. This approach could potentially facilitate testing multiple windows for sensitivity, clinical relevance, and variability, allowing the proposal of the most descriptive pAUC metrics.

## 5. Conclusions

Appropriate cut-off points for pAUC estimation in LAI product development need to be considered in a case-by-case scenario. Ideal pAUC metrics should offer insight into clinically relevant PK windows in the dosing interval without creating unmanageable variabilities. High variabilities of partial AUCs could lead to unnecessarily complicated BE study designs. This is especially important for LAI products, where multiple administrations often require patient populations in the investigation of BE due to their long dosing intervals. In such cases, survival and recruitment challenges for long and strenuous studies may render BE studies potentially unfeasible, which eventually prevents generic products from entering the market.

## Figures and Tables

**Table 1:** Summary of the requirements of pAUCs according to the FDA and EMA.

FDA [9]	EMA [10]
→For “*modified-release products in which the different phases of release correspond to a clinical effect*” and →For various reasons as described in specific PSGs	→For products with *low accumulation* along with the traditional PK metrics (Cmax, AUC_0-τ_) and →For various reasons as described in specific PSGs

**Table 2:** Mean pAUCs (±SD) and intersubject variability (%) after a single application of long-acting buprenorphine 192 mg in healthy volunteers (Buvidal^®^) (n = 13). Data from [18].

pAUC	Mean AUC (ng × days/mL) (±SD)	Intersubject Variability (%)
pAUC_0–3d_	15.14 (9.01)	59.51
pAUC_0–14d_	55.92 (20.77)	37.14

**Table 3:** Mean pAUCs (±SD) and intersubject variability (%) after a single application of long-acting naltrexone 380 mg (Vivitrol^®^) (n = 12). Data from [33].

pAUC	Mean AUC (ng × days/mL) (±SD)	Intersubject Variability (%)
pAUC_0–10d_	47.53 (18.84)	39.64
pAUC_10–28d_	71.78 (32.45)	45.22

**Table 4:** Mean pAUCs (±SD) and intersubject variability (%) after a single application of prolonged-release lanreotide 90mg (Somatuline Autogel^®^) (n = 10). Data from [47].

pAUC	Mean AUC (ng × days/mL) (±SD)	Intersubject Variability (%)
pAUC_0–7d_	33,458 (17,623)	52.71
pAUC_7–28d_	79,825 (51,176)	64.11
pAUC_28-td_	147,093 (160,237)	108.90

**Table 5:** Mean pAUCs (±SD) and intersubject variability (%) after a single application of leuprolide 1-month 3.75 mg (n = 24). Data from [78].

pAUC	Mean AUC (ng × h/mL) (±SD)	Intersubject Variability (%)
pAUC_24h-t_	273.10 (53.51)	19.59
pAUC_48h-t_	266.20 (50.76)	19.07
pAUC_72h-t_	262.10 (48.07)	18.34
pAUC_5d-t_	254.3 (15.48)	60.88
pAUC_7d-t_	25.88 (13.99)	62.00

**Table 6:** Sample size estimation for the parallel and crossover BE study design, according to variability (assuming T/R ratio of 90–111%, 80% power).

Variability (%) *	Parallel	Crossover
20	36	37
30	78	79
50	200	201
80	442	443
100	619	620

* Intersubject variability for parallel study designs and intrasubject variability for crossover designs.

**Table 7:** Sample size estimation and BE limits for the fully replicate crossover BE study design, according to variability and regulatory agency (assuming T/R ratio of 90–111%, 80% power).

	FDA		EMA	
Intrasubject Variability (%)	Sample Size	BE Limits	Sample Size	BE Limits
20	24	80–125%	18	80–125%
30	32	80–125%	34	80–125%
50	24	If S_WR_ is ≥0.294: the 95% upper confidence bound for ${\left({\overset{ˉ}{\Upsilon }}_{T}-{\overset{ˉ}{\Upsilon }}_{R}\right)}^{2}-\vartheta S_{WR}^{2}$ must be ≤0 and the point estimate of the test/reference geometric mean ratio must fall within [0.80, 1.25]	28	69.84–143.19%
80	28		50	69.84–143.19%
100	40		68	69.84–143.19%
